# Semiannual seasonality of COVID-19 with alternating peaks in winter and summer

**DOI:** 10.3389/fpubh.2026.1877365

**Published:** 2026-06-26

**Authors:** Hao Zhang, Jian Wang

**Affiliations:** 1 School of Geography, Nanjing Normal University, Nanjing, China; 2 Jiangsu Center for Collaborative Innovation in Geographical Information Resource Development and Application, Nanjing, China; 3 School of Geography, Jiangsu Second Normal University, Nanjing, China

**Keywords:** COVID-19, cycle analysis, ICEEMDAN, seasonality, temporal patterns

## Abstract

**Objective:**

To examine the presence of seasonality and characterize the temporal epidemic patterns of COVID-19 incidence during the main pandemic period.

**Methods:**

The Improved Complete Ensemble Empirical Mode Decomposition with Adaptive Noise (ICEEMDAN) method was used to systematically analyze COVID-19 incidence time series during the main pandemic period (March 1, 2020–February 28, 2023) from global, hemispheric, and key national perspectives.

**Results:**

Eight distinct epidemic waves occurred worldwide during the study period. The temporal pattern exhibited alternating peaks in winter and summer, corresponding to a semiannual cycle identified through time-series decomposition. This “winter–summer dual-peak” pattern was consistently observed across multiple spatial scales, although with regional variability in amplitude and timing.

**Conclusion:**

COVID-19 exhibited a clear semiannual seasonal pattern, differing from the typical unimodal winter dominance observed in many respiratory infectious diseases. This pattern may be associated with seasonal variations in environmental conditions. These findings may contribute to understanding the temporal dynamics of COVID-19 under normalized conditions and may provide reference for future studies on emerging respiratory infectious diseases.

## 1 Introduction

The acute emerging infectious disease caused by Severe acute respiratory syndrome coronavirus 2 (SARS-CoV-2), known as Coronavirus Disease 2019 (COVID-19), has posed an unprecedented threat to human health and has profoundly affected the global economy, social order, and international relations (1, 2). Although the World Health Organization (WHO) declared in May 2023 that COVID-19 no longer constitutes a “Public Health Emergency of International Concern” (3), localized outbreaks continue to emerge, and concerns persist regarding its long-term impacts on health and daily life. As the pandemic transitions into a phase of normalization, identifying the temporal outbreak patterns has become essential for developing precise and effective control strategies.

Epidemics caused by respiratory viruses often demonstrate pronounced seasonality. For example, influenza viruses and respiratory syncytial virus (RSV) typically peak in winter—December to March in the Northern Hemisphere and June to August in the Southern Hemisphere (4). Among human coronaviruses (HCoVs), four strains associated with mild upper respiratory infections—HCoV-OC43, 229E, NL63, and HKU1—also predominantly circulate in winter or early spring, a seasonal pattern that has been confirmed in multiple countries, including the United States, Belgium, France, and Japan (5–16). In contrast, due to their limited transmissibility, no definitive evidence has been established for the seasonality of severe coronaviruses such as SARS-CoV-1 and MERS-CoV (17–20).

Against this background, whether COVID-19 will evolve into a disease with a seasonality like other respiratory viruses has become a key focus of current research. However, research findings remain highly inconsistent. Some researchers argue that COVID-19 exhibits a seasonal pattern. Sajadi et al. found that the early spread of COVID-19 was closely associated with climatic conditions, suggesting a potential seasonal trend (21). A Bayesian estimation study in temperate regions of Europe showed strong seasonal transmission signals across all 143 regions analyzed (22). Liu et al. found that 40–60% of COVID-19 cases in 10 countries across the Northern and Southern Hemispheres were associated with seasonality, with a more pronounced effect observed at higher latitudes (23). In contrast, some studies have proposed opposite perspectives. For example, Mitchell et al. based on time series analyses of global and national data from 14 countries, a argued that COVID-19 transmission exhibits a periodic fluctuation of approximately 4 months, resembling an endogenous oscillatory pattern (24). Callaway noted that in the future, COVID-19 may manifest as sporadic and recurrent small-scale waves rather than traditional seasonal surges (25). In addition, some studies have emphasized that the outbreak pattern of COVID-19 remains uncertain. For example, Merow et al. suggested that while some degree of seasonality may exist, factors such as viral mutations and vaccination complicate the accurate prediction of COVID-19 outbreak patterns (26). Murray et al. proposed that although COVID-19 may persist and display some seasonal characteristics, the emergence of new variants and fluctuating immunity levels increase the likelihood of its becoming a recurrent seasonal infection (27). Del Rio et al. also suggested that COVID-19 may eventually evolve into an endemic or seasonal epidemic, although many uncertainties remain in this evolutionary trajectory (28).

Given the inconsistencies among existing research findings, it is particularly urgent to determine whether COVID-19 exhibits seasonality. As the pandemic has progressed and data have continued to accumulate, it is now feasible to systematically examine its outbreak patterns from a temporal perspective on a global scale. Based on a systematic analysis of the temporal evolution of COVID-19, this study focuses on the main epidemic period (March 1, 2020–February 28, 2023) and employs the ICEEMDAN method to perform a multi-level periodic analysis at the global, hemispheric, and national scales. The objective is to reveal the temporal evolution patterns of COVID-19. This study contributes to a deeper understanding of the long-term dynamic evolution of COVID-19 and offers theoretical insights and policy guidance for addressing similar emerging respiratory infectious diseases.

## 2 Materials and methods

### 2.1 COVID-19 data

The daily confirmed COVID-19 case data used in this study were obtained from WHO (29). The daily incidence rate (IR) for each region was calculated by standardizing the number of confirmed cases according to the population size:

$$IR_{t}=\frac{NC_{t}}{P}\times 1000000$$

where *IR*_t_ is the incidence rate (per million) on day t, *NC*_t_ is the number of confirmed cases on day t, and *P* is the total population of the region.

Missing values in the daily case time series were filled using linear interpolation based on adjacent observations. Negative values and abnormal records, which mainly arose from retrospective corrections and reporting adjustments, were corrected according to updates released by the WHO and official national public health agencies. This procedure ensured the consistency and reliability of the final dataset. A 7-day moving average was additionally applied to reduce short-term fluctuations.

### 2.2 Methods

This study employed ICEEMDAN (30), an adaptive signal analysis method based on Empirical Mode Decomposition (EMD), to decompose the COVID-19 IR time series at the global level, in the Northern and Southern Hemispheres, and in selected key countries during the pandemic period (from March 1, 2020, to February 28, 2023). ICEEMDAN introduces adaptive noise and iterative sifting to mitigate mode mixing and ensure the extraction of meaningful Intrinsic Mode Functions (IMFs) from non-stationary and nonlinear time series. Each IMF represents an oscillatory component, with higher-order IMFs capturing slower, low-frequency variations and lower-order IMFs capturing rapid, high-frequency fluctuations; the residual trend represents the long-term trajectory of COVID-19 incidence. After decomposition, each IMF was analyzed using Fast Fourier Transform (FFT) to identify dominant quasi-periodic characteristics. By performing ICEEMDAN decomposition across global, hemispheric, and national datasets, the multi-scale framework allowed detection of both widespread and region-specific temporal patterns. Low-frequency IMFs were further analyzed to detect the semiannual cycle and investigate temporal variations across different regions.

To assess the robustness of the identified temporal patterns, we additionally conducted sensitivity analyses using traditional seasonal decomposition methods. Specifically, Seasonal-Trend decomposition using Loess (STL) (31) and classical seasonal decomposition (CSD) (32) were applied to the global and hemispheric COVID-19 incidence rate time series. These complementary approaches were used to verify the robustness of the results.

## 3 Results

### 3.1 Temporal evolution of COVID-19 IR

**Figure 1:**
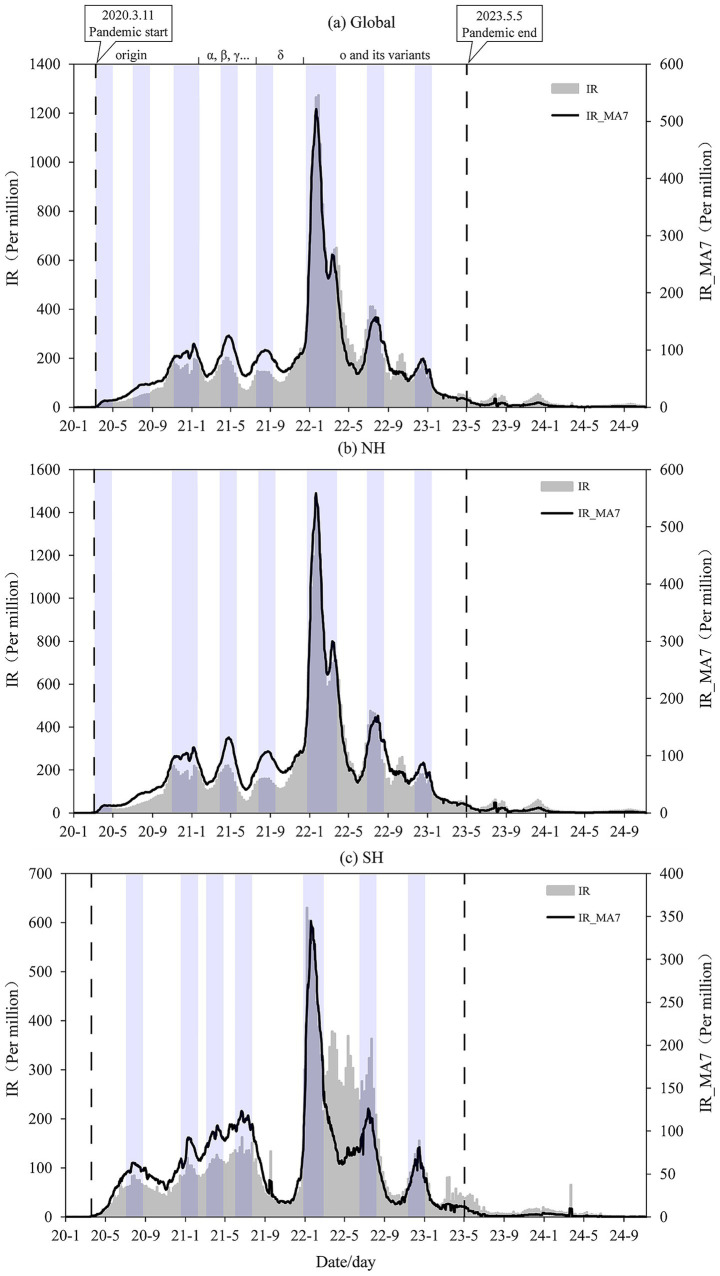
Global and hemisphere COVID-19 IR time evolution overview (IR, incidence rate; MA7, 7-day moving average. Purple areas represent peak pandemic periods, and the black solid line represents the 7-day moving average IR).

Figure 1 illustrates the predominant SARS-CoV-2 variants across different phases of the pandemic. From 2020 to February 2021, the original strain remained the dominant variant. Between March and July 2021, the Alpha (*α*), Beta (*β*), and Gamma (*γ*) variants gradually became dominant. From August to November 2021, the Delta (*δ*) variant became the predominant circulating strain. Since December 2021, the Omicron (*ο*) variant and its multiple sublineages have rapidly spread, becoming the dominant variants and continuing to prevail up to the present.

Over the course of the pandemic, the world experienced eight major waves of COVID-19 outbreaks (Figure 1; Table 1). Analysis of these peaks reveals regular patterns in the epidemic’s progression, with outbreaks frequently occurring around January, April, and August each year.

**Table 1:** Timing of COVID-19 IR peaks in the global and hemisphere regions.

No.	Global	NH	SH
1	Early April 2020	Early April 2020	
2	Early August 2020		Early August 2020
3	November 2020–February 2021	November 2020–February 2021	January 2021
4	Late April 2021	Late April 2021	Early April 2021
5	Late August 2021	Late August 2021	late June 2021
6	January to March 2022	January to March 2022	late January 2022
7	Late July 2022	Late July 2022	July 2022
8	Late December 2022	Late December 2022	Late December 2022

NH, Northern Hemisphere; SH, Southern Hemisphere.

It is worth noting that, due to the opposite seasons in the Northern and Southern Hemispheres, analyzing them separately is more conducive to revealing their distinct seasonal characteristics. The analysis showed that the first wave of the pandemic was primarily driven by the Northern Hemisphere, occurring during the transition from winter to spring, while the second wave was predominantly driven by the Southern Hemisphere during its winter season. Starting from the third wave (November 2020–February 2021), both hemispheres experienced epidemic peaks nearly simultaneously. The pandemic then exhibited a more synchronized global spread pattern, primarily characterized by alternating winter and summer outbreaks, with occasional smaller-scale peaks in spring and autumn. This “dual-peak” feature deviates from the traditional single-season pattern of respiratory viruses and mild coronaviruses, which predominantly occur in autumn and winter, highlighting the atypical temporal evolution of COVID-19.

With the end of the global pandemic, the overall epidemic situation worldwide eased, and some countries ceased continuous case reporting, resulting in a decline in the completeness of incidence data. However, localized outbreaks continued to occur. Available data indicated that the timing of epidemic fluctuations from May 2023 to December 2024 was roughly consistent with that of the previous 3 years (Supplementary Figure 1), suggesting a possible continuation of the original patterns.

### 3.2 COVID-19 incidence time series periodicity analysis

**Figure 2:**
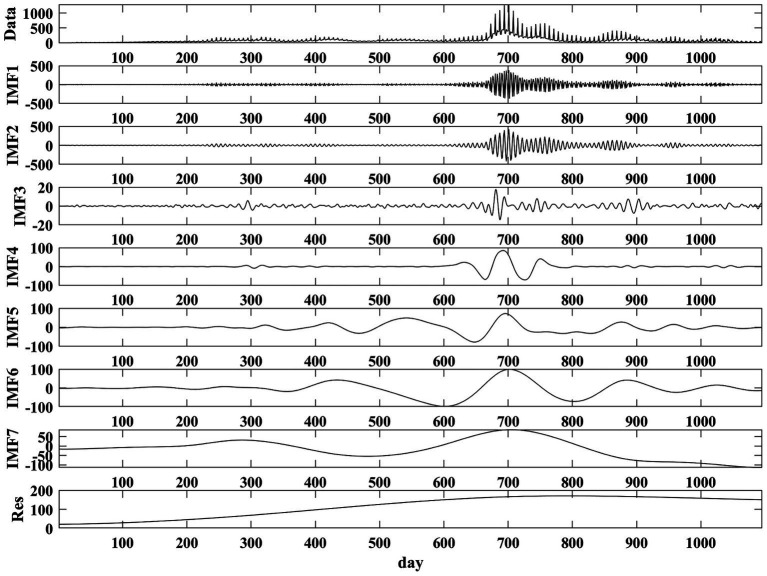
ICEEMDAN decomposition of the global time series from March 1, 2020, to February 28, 2023 (IMF: intrinsic mode function; Res: residue).

First, the 3-year time series for the globe, as well as the Northern and Southern Hemispheres, were decomposed using ICEEMDAN, yielding seven intrinsic mode functions (IMFs) and one trend component for each dataset (the global decomposition results are shown in Figure 2, with other results presented in Supplementary Figures 2 and 3).

**Figure 3:**
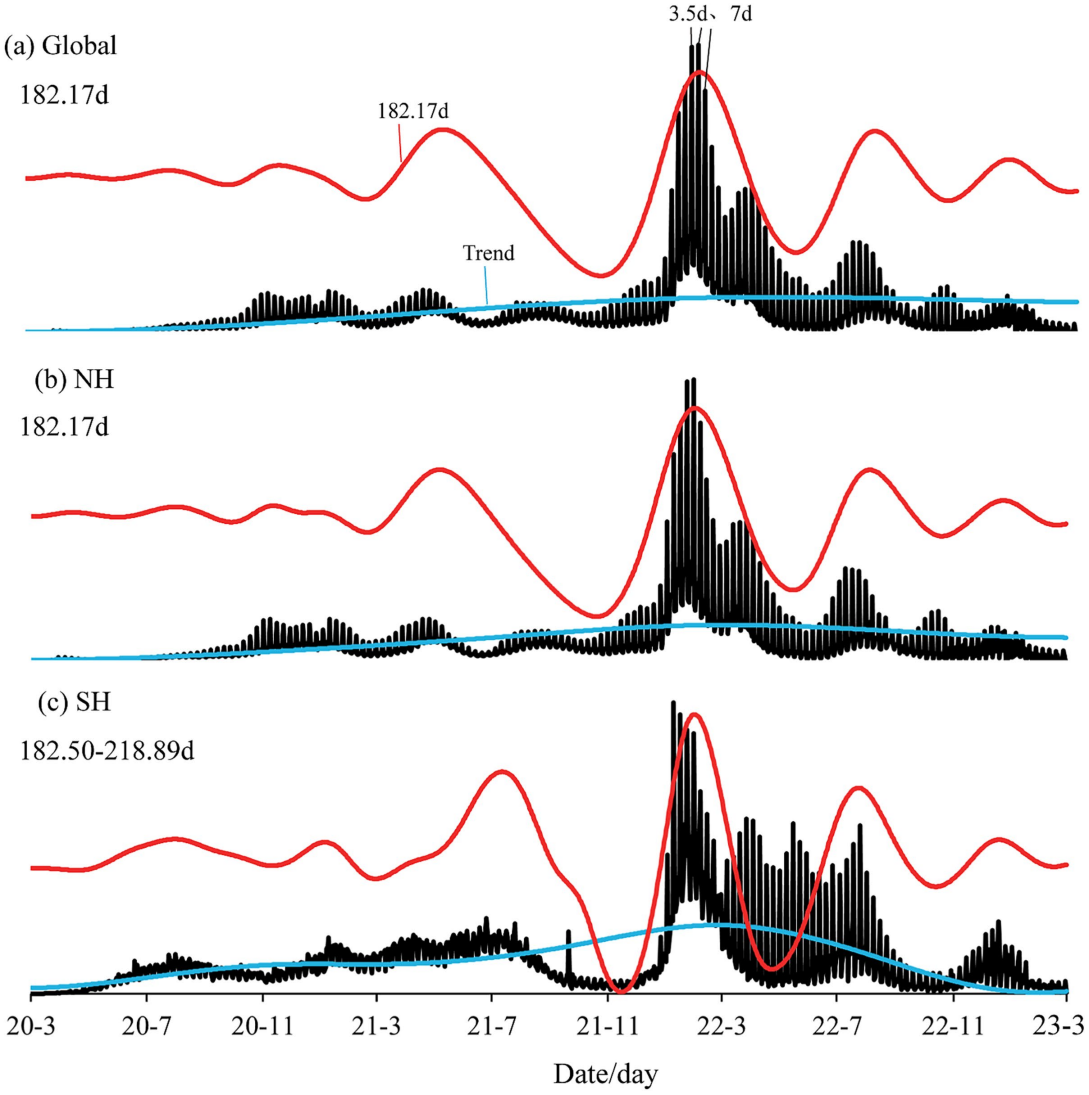
Original sequence, high-frequency component, and long-term trend of COVID-19 IR in the global and hemispheric time series from March 1, 2020, to February 28, 2023 (Black line represents the original sequence, red line represents the high-frequency component, blue line represents the long-term trend).

After spectral analysis of each IMF component, the results (Table 2) indicated that the quasi-periods for the globe, Northern Hemisphere, and Southern Hemisphere included approximately half-week (3.49–3.55d), 1 week (7.02–7.70d), 2 weeks (13.04–15. 61d), 2 months (60.83–64.41d), half-year (156.43–182.17–218.89d), and 1 year (365.00d). Among these, the variance contribution rates of the half-week and 1-week quasi-periods accounted for approximately 40.97–50.86%, the half-year quasi-period accounted for 15.27–25.49%, and the 1-year quasi-period accounted for 8.32–10.94%. Additionally, the IMF components were classified into high-frequency and low-frequency groups and merged separately to form a high-frequency component, a low-frequency component, and a trend term. Specifically, IMF1–IMF4 represented high-frequency cycles, whereas IMF5–IMF7 represented low-frequency cycles. The main periods in the high-frequency component were half-week and 1 week, while the low-frequency component predominantly exhibited the half-year cycle (Figure 3), which was a key factor influencing the medium- and long-term rhythms of the pandemic.

**Table 2:** Quasi-cycles of each IMF component in the global and hemispheric time series from March 1, 2020, to February 28, 2023.

IMF components	Component types	Global		NH		SH	
		VarR^a^/%	Cycles/d	VarR/%	Cycles/d	VarR/%	Cycles/d
IMF1	High	24.12	3.49	23.01	3.45	19.44	3.55
IMF2		26.74	7.02	25.51	7.31	21.53	7.70
IMF3		0.03	13.04	0.04	15.61	0.20	13.58
IMF4		2.01	64.41	2.33	64.29	0.21	60.83
IMF5	Low	12.13	156.43	11.19	156.43–168.17	5.17	99.55–104.10
IMF6		10.85	182.17	14.30	182.17	15.27	182.50–218.89
IMF7		8.51	365.00	8.32	364.43–437.20	10.96	365.00
Res	Trend	17.23	–	15.23	–	27.22	–

a Variance rate.

**Figure 4:**
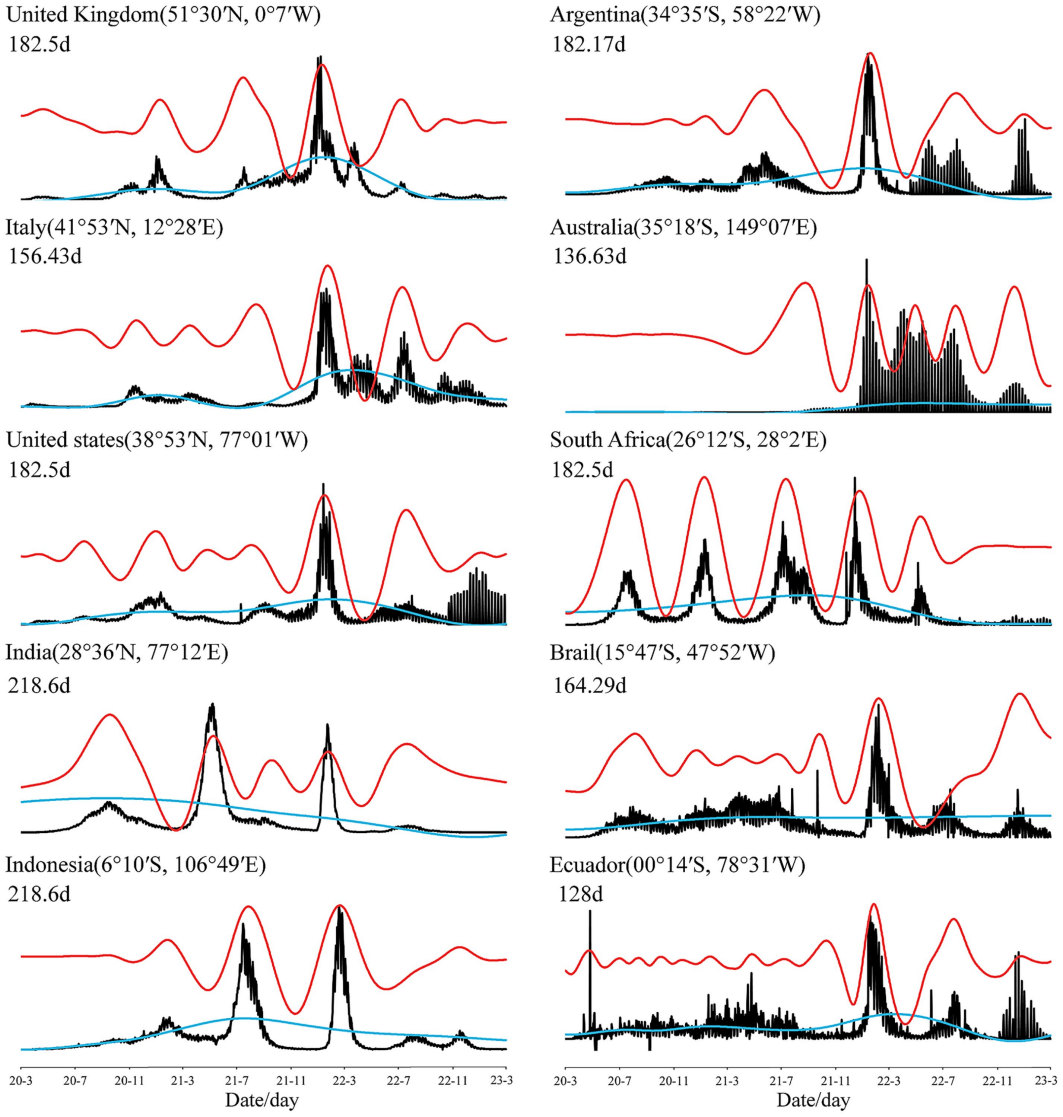
Original sequence, high-frequency component, and long-term trend of COVID-19 IR in 10 key countries from March 1, 2020, to February 28, 2023 (Black line represents the original sequence, red line represents the high-frequency component, blue line represents the long-term trend).

Furthermore, we applied the same analysis to 10 key countries in the Northern and Southern Hemispheres (five countries in each hemisphere). The results showed that these countries generally exhibited low-frequency cycles ranging from 4 to 7 months (128–218d), with the most prominent cycle remaining approximately half a year (Figure 4). Specifically, the cycle lengths were 128d for Ecuador and 136.63d for Australia; 156.43d for Italy and 164.29d for Brazil; 182.5d for the United Kingdom, the United States, and Argentina, 182.17d for South Africa; and 218.6d for India and Indonesia.

## 4 Discussion

**Figure 5:**
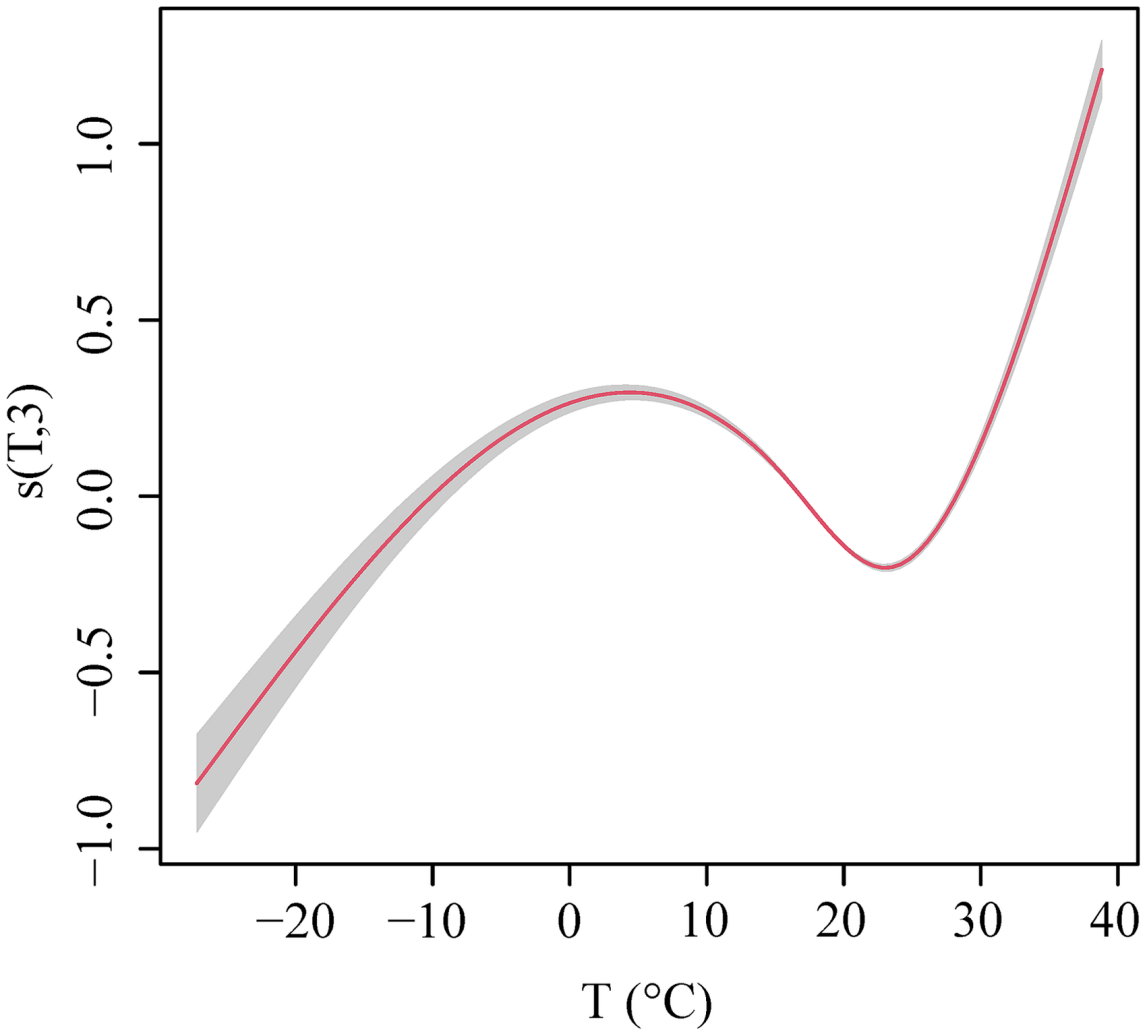
The nonlinear relationship between COVID-19 daily new cases and temperature (36).

By analyzing the temporal evolution of COVID-19 IR globally and across both hemispheres, we preliminarily identified a characteristic seasonal pattern featuring “dual peaks in winter and summer.” To further validate and quantify this phenomenon, the study employed the ICEEMDAN method to decompose time series data spanning from March 2020 to February 2023. The results revealed two main categories of periodic signals: first, a high-frequency cycle of approximately half a week to 1 week, likely driven by a combination of factors including reporting rhythms, testing frequency, behavioral patterns, and the virus’s incubation period (33); second, a low-frequency cycle of approximately half a year, which significantly corresponds to the phased outbreaks of the pandemic and suggests a semiannual seasonal pattern characterized by winter-summer dual peaks. It is worth noting that a similar semiannual cycle was also observed during the 2009 H1N1 pandemic, with related studies reporting this pattern in both China and Mexico (33–35). In addition, the seasonal pattern identified in this study aligns with our previous findings that COVID-19 infection risks significantly increased under both high and low temperature conditions (Figure 5) (36). This observation corroborates the semiannual cycle characterized by winter and summer peaks, further suggesting that this pattern may be closely linked to the seasonal fluctuations of multiple natural environmental factors, including temperature. These environmental variations may affect virus viability and human immune system functioning and therefore represent one possible explanation for the observed semiannual pattern.

Specifically, the high incidence of COVID-19 during winter and summer is closely associated with specific combinations of environmental factors, including temperature, humidity, and ultraviolet radiation. In winter, reduced solar radiation, lower temperatures, and weakened ultraviolet radiation—particularly in dry inland or high-latitude regions—lead to decreased air humidity and reduced moisture evaporation, resulting in abnormally dry air (37). This cold, dry, and low-ultraviolet environment provides ideal conditions for virus survival while simultaneously impairing human immune function, thereby significantly increasing the risk of infection. In humid climates, such as temperate oceanic zones, winter relative humidity tends to be relatively high. Under high humidity conditions, moist air causes the perceived temperature to be lower than the actual air temperature, which reduces human immunity; meanwhile, the humid environment facilitates the aggregation and enlargement of viral particles, shortening their airborne transmission distance, thus promoting viral spread and increasing infection risk (38). In this environmental context, although moist air may reduce the transmission distance of aerosol particles, viral transmission tends to be more direct and concentrated, thereby elevating the risk of infection.

In summer, with increased solar radiation, both temperature and ultraviolet intensity rise. In arid regions, the air generally becomes even drier. Although SARS-CoV-2 activity may decline under high temperature, dryness, and strong ultraviolet radiation, some studies have demonstrated that the virus retains its transmission capability (39). Meanwhile, the human immune system tends to be more vulnerable in hot and dry environments, generally exhibiting diminished immunity (40). Particularly under extreme heat, immune responses can be impaired, further elevating the risk of infection (41). Additionally, due to high temperatures, people tend to stay indoors, resulting in gatherings that enhance viral transmission opportunities and shorten transmission distances, thereby accelerating epidemic outbreaks. n humid regions, the combination of heat and moisture significantly raises the perceived temperature, which adversely affects human immunity (42). Although moist air increases atmospheric water content and reduces aerosol transmission distances, the elevated perceived temperature and humid conditions continue to adversely affect the human body. Under these circumstances, individuals become more susceptible to stress caused by temperature fluctuations, resulting in lowered immunity and an increased risk of SARS-CoV-2 infection (37).

In addition to the global semiannual pattern, our results also revealed notable regional heterogeneity. First, the timing of winter and summer peaks showed slight differences between the Northern and Southern Hemispheres, even though the seasons are opposite. Second, the amplitude of the seasonal peaks varied, with the Northern Hemisphere generally exhibiting larger fluctuations than the Southern Hemisphere. Third, the low-frequency cycles differed slightly among countries, ranging approximately from 4 to 7 months. These spatial variations may be influenced by multiple factors, including latitude, public health interventions, vaccination coverage, population mobility, and the timing of SARS-CoV-2 variant emergence.

It should also be acknowledged that the study period coincided with substantial public health interventions, including non-pharmaceutical measures, large-scale COVID-19 vaccination rollout, and the successive emergence of multiple SARS-CoV-2 variants. These factors undoubtedly had important impacts on COVID-19 transmission dynamics, particularly in terms of the magnitude and intensity of epidemic waves. However, their influence was mainly reflected in short-term fluctuations in transmission levels rather than in the long-term temporal structure identified in this study. Although viral variants may alter transmissibility and incubation periods, such effects primarily operate at relatively short temporal scales and are unlikely to substantially modify the low-frequency semiannual cycle detected in the present analysis.

### 4.1 Sensitivity analysis

To assess the robustness of the identified semiannual cycle, we examined its consistency across different spatial and temporal scales. ICEEMDAN-based analyses conducted at the global, hemispheric, and selected national levels, together with extended time series beyond 2023 for some countries, consistently identified a stable semiannual periodicity. Furthermore, traditional seasonal decomposition methods, namely STL and CSD, were applied to the global and hemispheric time series and yielded similar low-frequency (~6-month) patterns. The consistency of results across different datasets and analytical approaches further supports the robustness of the observed semiannual cycle. The corresponding results are presented in Supplementary Tables 1 and 2.

### 4.2 Strengths and limitations

This study has several strengths. First, it analyzed COVID-19 incidence over a long time span covering the main pandemic period, which allowed the identification of robust macro-scale temporal patterns. Second, a multi-scale analytical framework was applied by incorporating global, hemispheric, and selected national datasets, improving the consistency and comparability of the results across spatial levels. Third, the use of the ICEEMDAN method enabled effective decomposition of non-stationary time series and facilitated the identification of intrinsic oscillatory components and periodic characteristics.

However, several limitations should also be acknowledged. This analysis was conducted at global and national scales and therefore mainly captured macro-level temporal patterns of COVID-19 incidence. Future studies incorporating high-resolution spatiotemporal datasets are needed to further elucidate the underlying mechanisms driving the observed temporal patterns.

### 4.3 Conclusion

A clear semiannual seasonal pattern of COVID-19 incidence characterized by alternating winter and summer peaks was identified. These findings enhance the understanding of the temporal dynamics of COVID-19 and imply that, within routine public health surveillance, both winter and summer periods may represent phases of elevated transmission risk. In addition, they provide a useful reference for future studies on the temporal patterns and surveillance of emerging respiratory infectious diseases.

## Data Availability

Publicly available datasets were analyzed in this study. This data can be found here: https://covid19.who.int.
