# Supplementary material for: Semiannual seasonality of COVID-19 with alternating peaks in winter and summer
Source: Front Public Health. 2026 Jun 26;14:1877365. doi: 10.3389/fpubh.2026.1877365 (PMC13350458; doi:10.3389/fpubh.2026.1877365)
Supplement: Supplementary file 1 [file Table_1.DOCX]

Supplementary Material

# Supplementary Figures


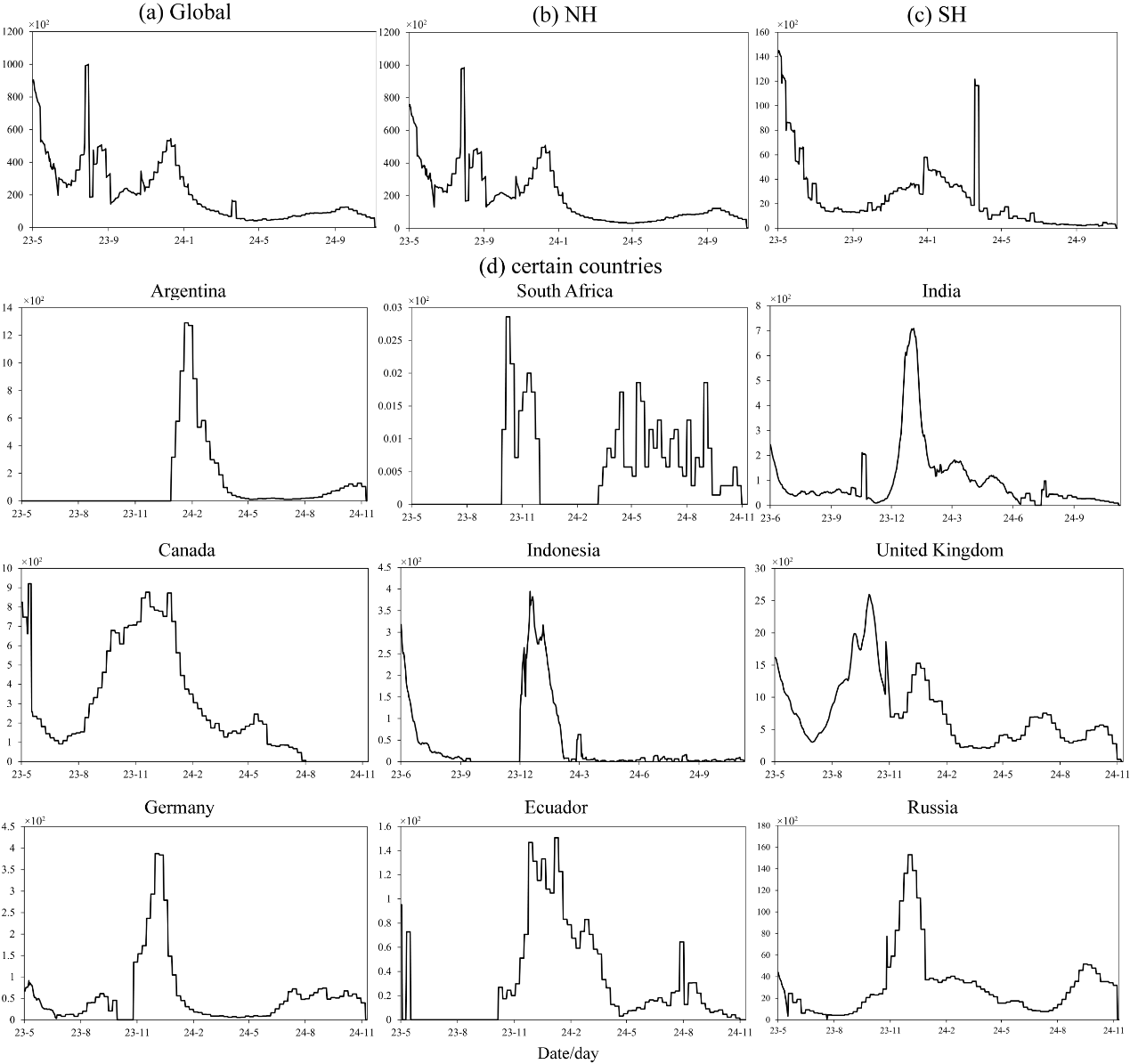
**Supplementary Figure 1.** Temporal Changes in COVID-19 Incidence Rates Across the World, Hemispheres, and Selected Countries After the End of the Global Pandemic


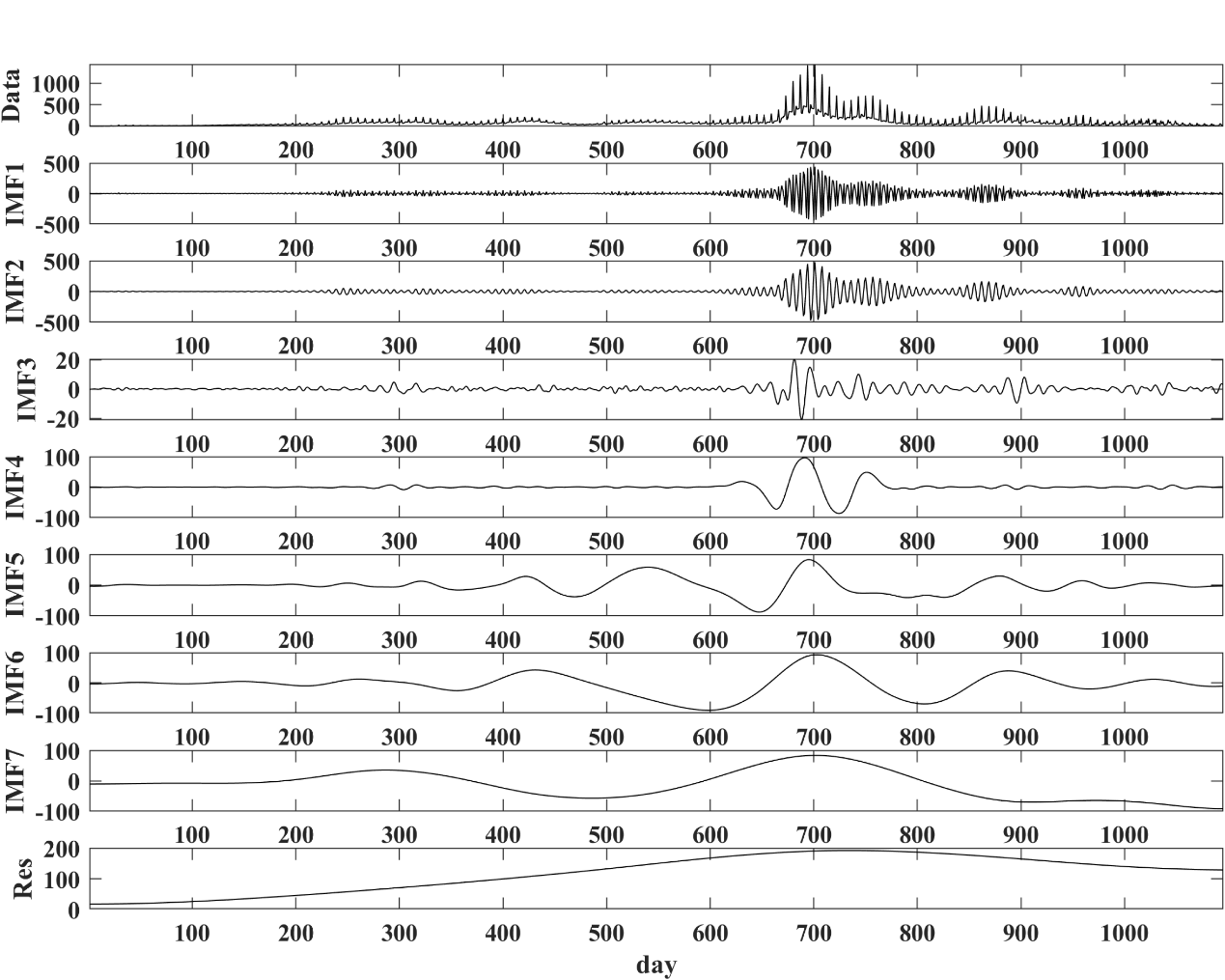
**Supplementary Figure 2.** ICEEMDAN Decomposition of the Northern Hemisphere Time Series from March 1, 2020, to February 28, 2023


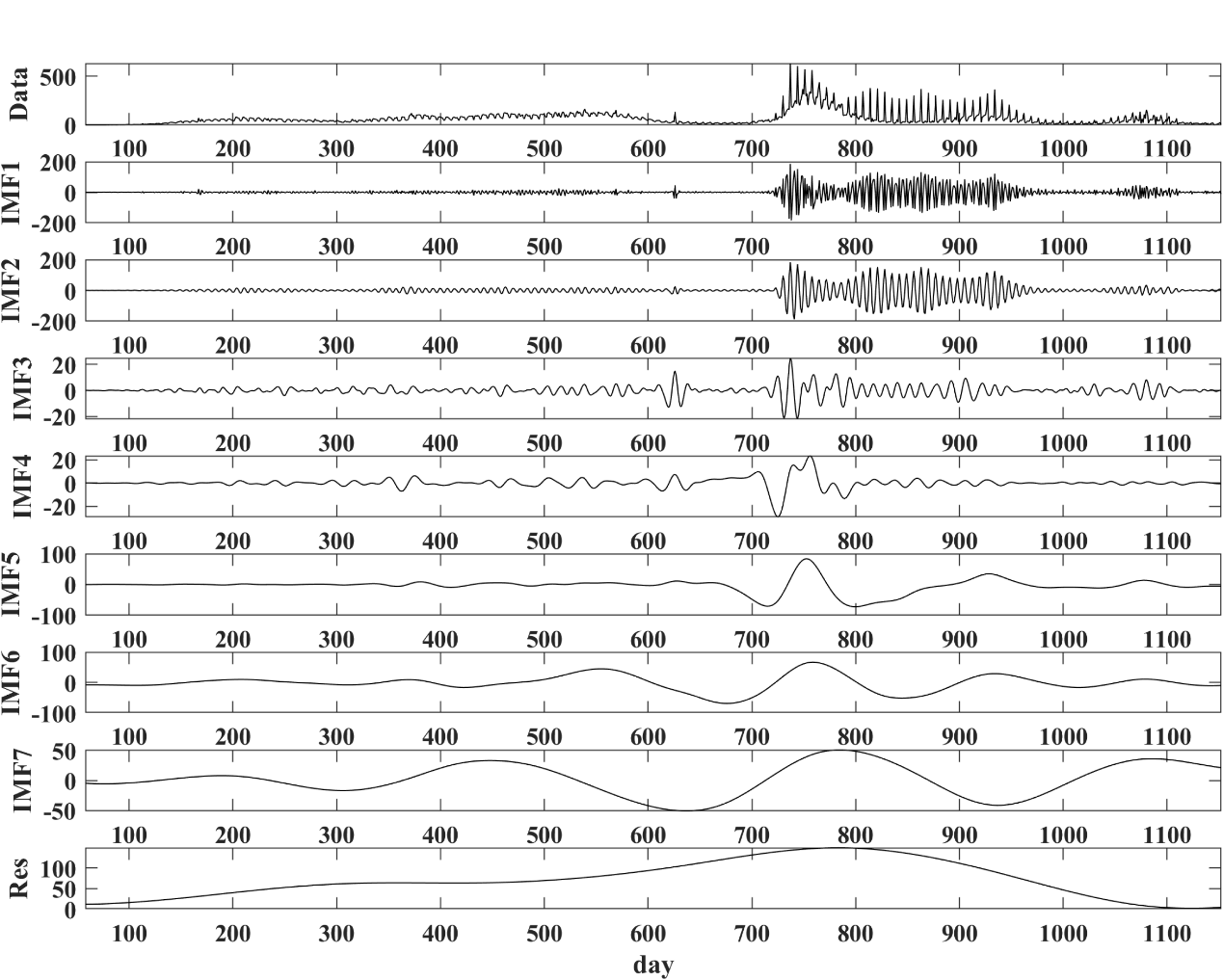
**Supplementary Figure 3.** ICEEMDAN Decomposition of the Southern Hemisphere Time Series from March 1, 2020, to February 28, 2023

# Supplementary Tables

**Supplementary Table 1.** Quasi-periods and spectral power of global and hemispheric COVID-19 incidence rates identified using Seasonal-Trend decomposition using Loess (STL)

|  | Global | | NH | | SH | |
| --- | --- | --- | --- | --- | --- | --- |
|  | Power | Cycles/d | Power | Cycles/d | Power | Cycles/d |
| 1 | 230.68 | 3.50 | 282.65 | 3.50 | 60.12 | 3.50 |
| 2 | 501.82 | 7.03 | 548.31 | 7.03 | 310.20 | 7.03 |
| 3 | 142.12 | 93.75 | 166.50 | 93.75 | 50.43 | 93.75 |
| 4 | **661.29** | **187.50** | 91.74 | 125.00 | **754.63** | **187.50** |
| 5 | **1015.76** | **375.00** | **651.17** | **187.50** | **413.42** | **375.00** |
| 6 |  |  | **1320.17** | **375.00** |  |  |

**Supplementary Table 2.** Quasi-periods and spectral power of global and hemispheric COVID-19 incidence rates identified using classical seasonal decomposition (CSD)

|  | Global | | NH | | SH | |
| --- | --- | --- | --- | --- | --- | --- |
|  | Power | Cycles/d | Power | Cycles/d | Power | Cycles/d |
| 1 | 467.28 | 3.50 | 586.61 | 3.50 | 95.38 | 3.50 |
| 2 | 134.27 | 6.85 | 161.58 | 6.85 | 525.99 | 7.03 |
| 3 | 979.10 | 7.03 | 1095.95 | 7.03 | 132.41 | 93.75 |
| 4 | 312.39 | 93.75 | 358.50 | 93.75 | 108.19 | 125.00 |
| 5 | **1193.34** | **187.50** | 159.42 | 125.00 | **1034.37** | **187.50** |
| 6 | **1729.02** | **375.00** | **1235.38** | **187.50** | **918.63** | **375.00** |
|  |  |  | **2165.57** | **375.00** |  |  |
